# How Does Emotional Context Modulate Response Inhibition in Alexithymia: Electrophysiological Evidence from an ERP Study

**DOI:** 10.1371/journal.pone.0051110

**Published:** 2012-12-05

**Authors:** Lei Zhang, Rong Ye, Fengqiong Yu, Zhaolun Cao, Chunyan Zhu, Zhu Cai, Panpan Hu, Hui Pu, Kai Wang

**Affiliations:** 1 Laboratory of Cognitive Neuropsychology, Department of Medical Psychology, Anhui Medical University, Hefei, Anhui Province, People’s Republic of China; 2 Department of Neurology, The First Hospital of Anhui Medical University, Hefei, Anhui Province, People’s Republic of China; Catholic University of Sacred Heart of Rome, Italy

## Abstract

**Background:**

Alexithymia, characterized by difficulties in identifying and describing feelings, is highly indicative of a broad range of psychiatric disorders. Several studies have also discovered the response inhibition ability impairment in alexithymia. However, few studies on alexithymic individuals have specifically examined how emotional context modulates response inhibition procedure. In order to investigate emotion cognition interaction in alexithymia, we analyzed the spatiao-temporal features of such emotional response inhibition by the approaches of event-related potentials and neural source-localization.

**Method:**

The study participants included 15 subjects with high alexithymia scores on the 20-item Toronto Alexithymia Scale (alexithymic group) and 15 matched subjects with low alexithymia scores (control group). Subjects were instructed to perform a modified emotional Go/Nogo task while their continuous electroencephalography activities were synchronously recorded. The task includes 3 categories of emotional contexts (positive, negative and neutral) and 2 letters (“M” and “W”) centered in the screen. Participants were told to complete go and nogo actions based on the letters. We tested the influence of alexithymia in this emotional Go/Nogo task both in behavioral level and related neural activities of N2 and P3 ERP components.

**Results:**

We found that negatively valenced context elicited larger central P3 amplitudes of the Nogo–Go difference wave in the alexithymic group than in the control group. Furthermore, source-localization analyses implicated the anterior cingulate cortex (ACC) as the neural generator of the Nogo-P3.

**Conclusion:**

These findings suggest that difficulties in identifying feelings, particularly in negative emotions, is a major feature of alexithymia, and the ACC plays a critical role in emotion-modulated response inhibition related to alexithymia.

## Introduction

Alexithymia has been defined as a reduced ability to identify, decode, and communicate feelings or emotional aspects of social interaction [1], [2]. It was originally described by Sifneos in patients with psychosomatic and psychiatric disorders [3]. More recently, alexithymic characteristics have been observed in nonclinical populations [4]; 1 study reported these characteristics in approximately 10% of the examined healthy individuals [5]. In both clinical and nonclinical populations, alexithymia is viewed as a personality trait, although its degree differs in different populations [6]. The alexithymia construct is most widely assessed by the Toronto Alexithymia Scale [7], a well-validated self-report questionnaire [8], [9], [10], using which 3 main facets, difficulties in identifying feelings, difficulties in describing feelings, and externally oriented thinking or preoccupation with details of external events, can be differentiated.

Response inhibition, defined as the ability to suppress inappropriate thoughts and actions, is an important component of human behavior. The Go/Nogo paradigm is an objective and direct measurement of the general efficiency of fast information processing, including inhibition processing. More than 2 decades of research confirm the result that the Go–Nogo paradigm typically elicits 2 components, the frontal N2 (200–400 ms) and frontocentral P3 (300–500 ms), whose amplitudes are larger in Nogo trials than in Go trials [11], [12]. The Nogo-N2 and Nogo-P3 components are currently thought to represent different aspects of response inhibition. The Nogo-N2 has also been linked to effortful attention, detection of response conflict, and action monitoring [13], [14]. On the contrary, Nogo-P3 has been primarily related to the inhibitory [15], [16]. Thus, it appears there is consistent evidence that the N2 and P3 represent different underlying processes in the Nogo–Go task (response conflict and response inhibition, respectively).

As is known, emotion usually interacts with cognition, and mood fluctuations may influence individuals’ ability to inhibit inappropriate responses in life settings [17], [18]. There is evidence that negative affectivity, especially depressive mood, is associated with impaired emotional awareness as well as impaired executive function [19]. However, using event-related potentials (ERPs), Albert showed that withholding a prepared response to positive emotion is more difficult and consumes greater inhibitory resources than withholding a prepared response to negative emotions [20]. Recently, the neural mechanisms underlying the interaction of emotion and response inhibition have been investigated extensively. This interaction is well reflected in several prefrontal regions [21], , including the anterior cingulate cortex (ACC) [23]. There is evidence that alexithymia, which is crucial in affect regulation, is linked to ACC and mediofrontal activity during emotional stimuli processing [24]. Functional neuroimaging studies have reported that alexithymic individuals exhibit decreased activation of the dorsal ACC in response to painful pictures [25] and emotional movie clips [26]. However, it remains unclear whether the role of this region in emotion-modulated response inhibition is related to alexithymia. Considering these results, it is necessary to examine the response inhibition differences during various emotional contexts and whether the ACC plays a role in alexithymia. To examine this, a modified version of the Nogo–Go task was used; this version required inhibition of prepotent responses to neutral cues during 3 different emotional contexts (negative, neutral, and positive) generated by pictorial backgrounds. As described, Nogo-N2, Nogo-P3, Nogo–Go N2, and Nogo–Go P3 are associated with different aspects of response inhibition. Therefore, our analyses focused on these components.

The present study aimed to examine the difference of Nogo-N2, and Nogo-P3 during 3 emotional contexts using ERPs between alexithymic individuals and controls in conjunction with a source-localization technique.

## Methods

### Participants

Two hundred students from Anhui Medical University in grade three were selected for alexithymia using the Chinese version of the Toronto Alexithymia Scale (TAS-20) [27]. The participants who scored higher than 59 were in alexithymic group, and who scored lower than 41 were in control group. All participants also met the following exclusion criteria: (a) no current substance (including alcohol) abuse. (b) no depressive disorder or anxiety disorder. (scores of the Self-Rating Depression Scale (SDS) of ≤41, and scores of the Self-Rating Anxiety Scale (SAS)≤40 ); (c) no demonstrable brain disease (such as epilepsy, schizophrenia, brain injury, or head trauma); The final sample included 15 students who scored higher than 59 in alexithymic group (mean = 64.93; SD = 7.32) and 15 students who scored lower than 41 in control group (mean = 36.06; SD = 3.34). Their ages ranged from 20 to 22 years. The proportion of females to males was similar in the two groups (Table 1). The study was approved by The Ethics Committee of Anhui Medical University. All participants gave written consents and got monetary rewards.

**Table 1:** Demographic data of alexithymic and control group.

	Alexithymics(n = 15)	Controls(n = 15)
Age (years)	21.2(0.46)	21.1(0.26)
Gender (male/female)	15 m/18f	16 m/14f
Education (years)	16.19(0.40)	16.13(0.34)
SDS	33.12(4.57)	31.94(3.12)
SAS	28.72(2.61)	27.64(2.23)
TAS-20 (score)	64.93(7.32)	36.06(3.34) *

* p<.05.

### Stimuli and Procedure

**Figure 1:**
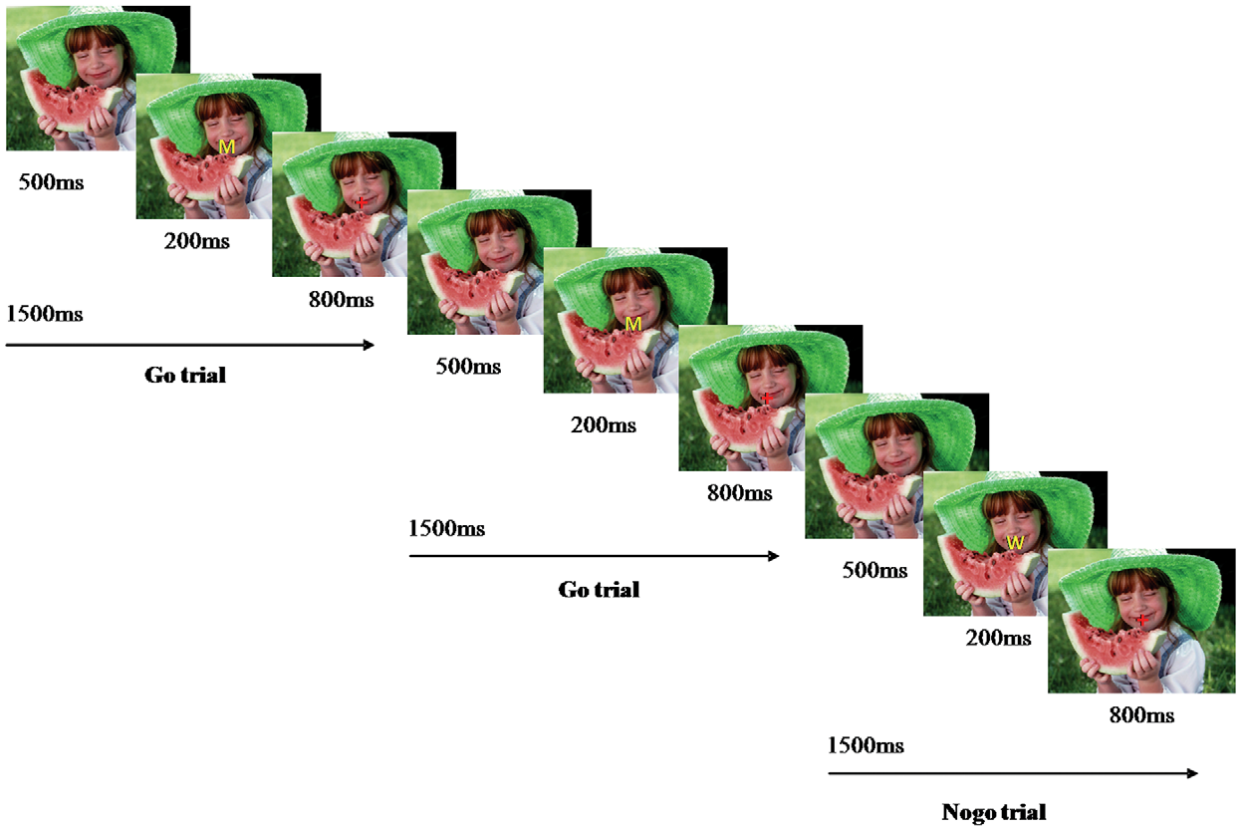
Examples of Go and Nogo trials in a positive context block.

The stimuli consisted of 2 capital letters (“M” and “W”) and 15 pictures used as background contexts (5 positive, 5 neutral, and 5 negative). Letters were colored in yellow so that they were clearly highlighted in the background. Pictures were taken from the International Affective Picture System (IAPS) [28] and used as background contexts. Pictures were selected based on their IAPS scores in arousal and valence. Moreover, each participant of two groups assessed each picture valence and arousal level and filled out a bi-dimensional scaling test for each picture. Table 2 shows the mean and standard deviations for the 3 types of emotional context (negative, neutral, and positive). In addition, pictures of each emotional context were matched in mean spatial frequency and luminance [29]. Subjects were instructed to press a button as quickly and accurately as possible whenever the letter “M” (Go) was presented and to withhold pressing the button when presented with the letter “W” (Nogo). They were also instructed to look continuously at the center of the screen and to avoid blinking during the block runs, in order to control the interference due to eye movement. Between each experimental block (about 3 min), subjects were allowed to rest. Participants performed the task during 3 different emotional context backgrounds: negative, neutral, and positive. The order across subjects was counterbalanced in a Latin-square design. Each emotional context contained 200 letters (150 Go and 50 Nogo) presented in 3 blocks. Each trial began with the presentation of the letter “M” or “W” (200 ms) followed by a fixation cross (800 ms), and the next letter appeared 500 ms later. Go and Nogo trials were presented in semi-random order to avoid consecutive presentation of 2 Nogo trials. Both the letter and fixation cross were superimposed on the center of the background picture (Fig. 1). Before beginning the experiment, subjects completed a practice block of 20 trials (15 Go and 5 Nogo) to ensure understanding of the task instructions. Moreover, all participants provided subjective ratings for each emotional context in order to discriminate the valence from arousal effects on neural and behavioral data.

**Table 2:** Means and standard deviations of valence (1, negative, to 7 positive) and arousal (1, calming, to 7, arousing) assessments given by the two groups to the three types of emotional context (Negative, Neutral, and Positive).

	Negative context*		Neutral context**		Positive context***	
	Valence	Arousal	Valence	Arousal	Valence	Arousal
Alexithymics	2.64(0.87)	4.3(1.69)	4.67(0.67)	2.26(1.37)	6.08(0.64)	3.44(1.76)
Controls	2.34(0.43)	5.15(0.36)	4.3(0.36)	3.14(1.24)	6.23(0.57)	4.04(1.47)

After asterisks, the IAPS code of employed pictures is provided.

* 1090 6210 6840 9320 9912.

** 2840 5510 7002 7009 7150.

*** 1999 2165 5831 5849 7325.

### EEG Recording

The electroencephalography (EEG) was recorded from 64 Ag-AgCl electrodes placed on the scalp according to the extended International 10/20 system (Waveguard64 cap, Cephalon A/S). Vertical electro-oculograms (EOGs) were recorded supraorbitally and infraorbitally at the left eye. Horizontal EOG was recorded as the left versus right orbital rim. The EEG activity was recorded using a left mastoid reference electrode and re-referenced off-line to the mean of bilateral mastoid electrodes. All electrode impedances were maintained below 5 kΩ. EEG and EOG activity was amplified with a DC 0.01-100 Hz band-pass and continuously sampled at the rate of 512 Hz (64 channel high-speed amplifier, Advanced Neuro Technology, Enschede, The Netherlands).

### Behavioral Data Analysis

The performance data of emotional-context Go/Nogo task including reaction times of go stimulus, errors of omission (no response to go trials) and commission (response to nogo trials) were submitted to repeated-measures analyses of variance (ANOVAs). The between-subject factors were alexithymic and control groups and the emotional contexts (positive, neutral, negative) were submitted as within-subject factors. For group differences in demographical and scale data, independent t-test of two samples was performed.

### EEG Data Analysis

Continuous EEG data were subsequently submitted to an open source toolbox EEGLAB [30] running in MATLAB 7.1 (The Mathworks) environment for off-line analysis. Data were first visually screened for any noisy part then high-pass filtered using the standard of 1 Hz. Eye blinks and eye movements artifacts were removed using a validated method based on an independent component analysis [31] implemented in EEGLAB. Filtered and artifact-free EEG data were time locked to the onset of word stimuli, and were divided into 1200-ms epochs beginning 200 ms before stimulus onset. Epochs containing the amplitudes beyond the range of ±100 µV were deleted. The time-domain averaging procedure was performed across trials for each subject and condition. Time windows of N2 and P3 components of obtained average waveforms were established based on the grand averages potentials of each task conditions. Within those time windows, the greatest negative and positive deflections from baseline to peak are identified as latencies for N2 and P3 respectively. Finally, the interval of N2 component was 250∼350 ms and the interval of P3 was 450∼550 ms. The following 9 electrode sites F3, C3, P3, Fz, Cz, Pz, F4, C4, P4 (3 frontal sites, 3 central sites and 3 parietal sites) were further selected for statistical analysis of N2 and P3 components. All statistical analyses described below were carried out using the 16.0 SPSS software package. We focused on task type (Go, Nogo) and group (alexithymia and control) and valence (negative, neutral, and positive) interaction effects for the averaged amplitudes and latency by conducting repeated-measures ANOVA. The degrees of freedom of the F-ratio were corrected according to the Greenhouse – Geisser method.

### Standardized Low-resolution Electromagnetic Tomography for Source-localization Analysis

Source localization was performed for components that differed between the 2 groups using standardized low-resolution electromagnetic tomography (sLORETA) [32]. sLORETA is a method for estimating cortical generator localization at specific time windows. sLORETA calculates the standardized current density at each of 6,239 voxels in the grey matter and hippocampus of the Montreal Neurological Institute brain. The voxel-based sLORETA-images (6,239 voxels at a spatial resolution of 5 mm) [32] were used to compare P3 amplitudes of the Nogo–Go difference wave between 2 the groups by using the sLORETA-built-in voxelwise randomization tests (5,000 permutations) based on statistical non-parametric mapping [33], corrected for multiple comparisons. Voxels with significant differences (P<0.01) were located in specific brain regions.

## Results

### Demographic and Behavioral Data

There were no significant differences between the 2 groups in age, gender, education, SDS, SAS (Table 1). Moreover, results from valence and arousal assessment also showed no significant differences between alexithymia and control group (Table 2).

The ANOVAs were used on RTs of go stimulus and errors of omission and commission. Data with the response time above 1,500 ms or below 150 ms were omitted from the analyses. There were no significant effect of emotional contexts, intergroup differences or emotion group interaction in RTs and errors of omission and commission (Table 3).

**Table 3:** Means and standard deviations of reaction times (RTs) of Go stimuli and omission/commission errors under each emotional context (Negative, Neutral, and Positive) in two groups.

	Negative context		Neutral context		Positive context	
	Alexithymics	Controls	Alexithymics	Controls	Alexithymics	Controls
Go RTs (ms)	383.78(37.56)	384.87(48.55)	390.22(46.27)	381.89(58.07)	393.90(47.54)	389.44(73.92)
Commission errors	5.62(4.41)	4.84(3.67)	6.85(5.74)	5.77(4.95)	5.77(4.59)	5.15(3.65)
Omission errors	0.85(1.14)	0.53(1.13)	0.77(1.48)	0.08(0.28)	0.85(1.52)	0.85(1.14)

### Event-related Potential Data

#### Original event-related potentials

**Figure 2:**
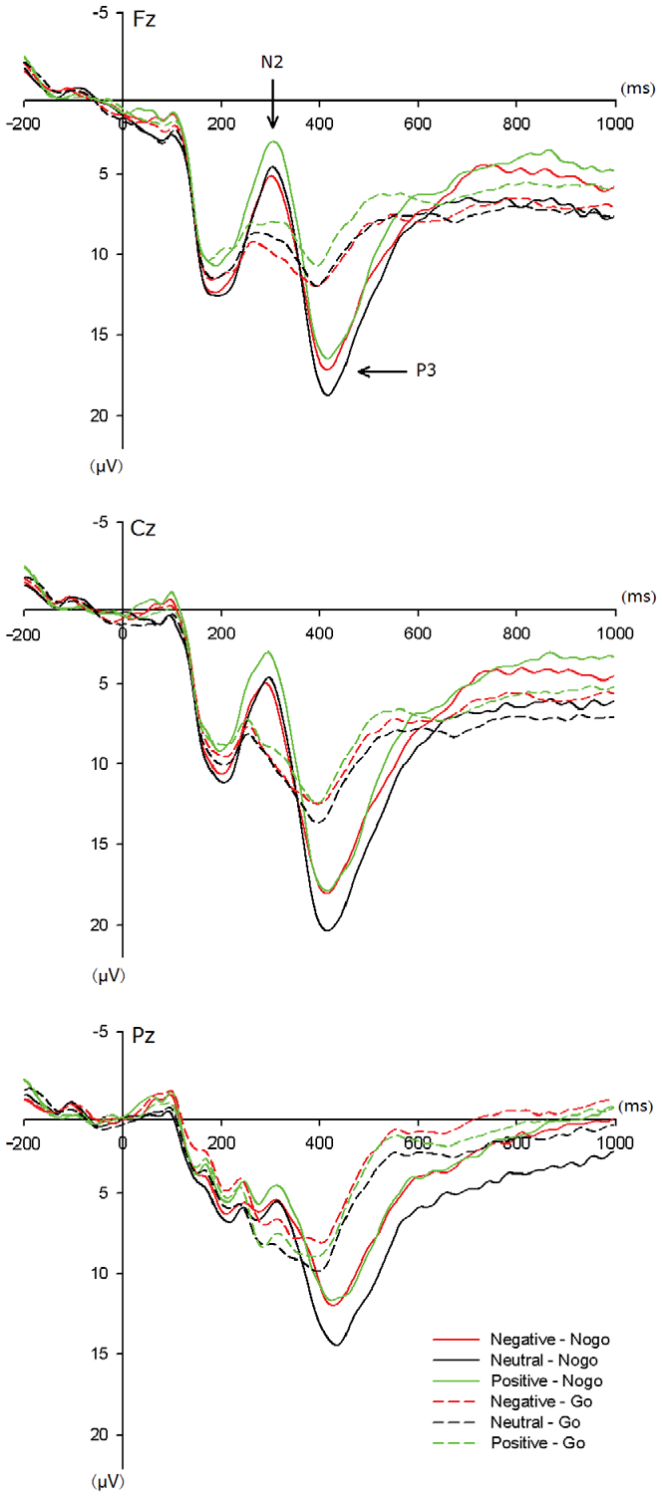
The averaged ERP evoked by Nogo (solid lines) and Go (dashed lines) stimuli during negative (red lines), neutral (black lines) and positive (green lines) emotional conditions at Fz, Cz and Pz.

The results of repeated-measures ANOVA on the averaged amplitudes of N2 and P3 components showed significant within-subject main effects of task [N2: *F*(1,29) = 18.83, p<0.001; P3: *F*(1, 29) = 107.51, p<0.001], valence [N2: *F*(2,87) = 6.15, p = 0.004; P3: [*F*(2, 87) = 6.74, p = 0.003] and electrode sites [N2: *F*(2,87) = 37.56, p<0.001; P3: *F*(8, 261) = 81.11, p<0.001]. The group effect of N2 and P3 did not reach the significance. No significant effects of latencies were observed for both N2 and P3 components (see Fig. 2).

Furthermore, we found a significant within-subject interaction of valence, task and electrode site in N2 stage [*F*(2,87) = 3.75, p = 0.001]. This result showed that in Nogo condition N2 amplitude recorded in fronto-central electrodes was larger during positive emotional context than other two emotional contexts.

More importantly, we also found a significant between-subject interaction of group, valence and task [*F*(2,87) = 77.62, p = 0.024] in P3 stage. For clearly illustrate this interaction effect, we calculated the Nogo–Go difference waves to observe the dissimilarity between alexithymia and control groups.

#### Nogo–Go difference event-related potentials

**Figure 3:**
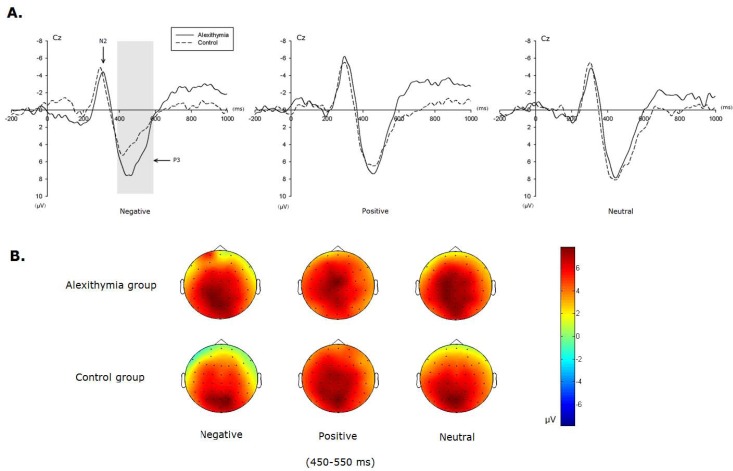
The difference ERPs of Nogo minus Go tasks between two groups during 3 emotional conditions at Cz. A. Difference waveforms recorded at Cz site under 3 emotional contexts (negative, positive and neutral conditions); B. 2-D topographic display of mean voltage of the P3 difference waves (time interval: 450–550 ms) for both alexithymia and control group during 3 emotional conditions.

As shown in Figure 3, the Nogo–Go difference waves and compatible scalp topographies clearly illustrate the dissimilarity between the 2 groups. The repeated-measures ANOVA on Nogo–Go N2 amplitudes as a dependent measure revealed a significant main effect of valence [*F*(2,87) = 3.59, p<0.05] and electrode sites [*F*(8,261) = 12.74, p<0.001]. This result reflected the higher N2 amplitudes over frontal electrodes compared to central and posterior ones, and more negative Nogo–Go N2 amplitudes during the negative emotional context in alexithymia group compared to the control group.

The repeated-measures ANOVA on P3 amplitudes of the Nogo–Go difference wave revealed no significant main effect of valence [*F*(2,87) = 1.40, p>0.05]. However, the interaction effect between group and valence was significant [*F*(2,87) = 3.77, p<0.05]. For alexithymic individuals, there were no significant main effects of valence [*F*(2, 42) = 0.65, p>0.05]. However, for the control group, there were significant main effects of valence [*F*(2, 42) = 4.03, p<0.05]. Importantly, this indicates significantly different amplitudes between alexithymia and control groups only in response to negative context [*F*(1, 29) = 4.47, p<0.05]. The amplitude during negative emotional conditions was much larger for the alexithymic group than for the control group (Fig. 3A). The topographies of P3 difference waves under 3 emotional contexts also demonstrated the similar result to amplitude analysis (Fig. 3B). The electric field distribution of Nogo-Go difference waves during the time window of 450 to 550 ms showed the P3 component mainly centered in the central and posterior part of the scalp. Compared with the control group, the alexithymic group exhibited more topographic difference in negative condition than other emotional contexts.

#### Source-localization data

**Figure 4:**
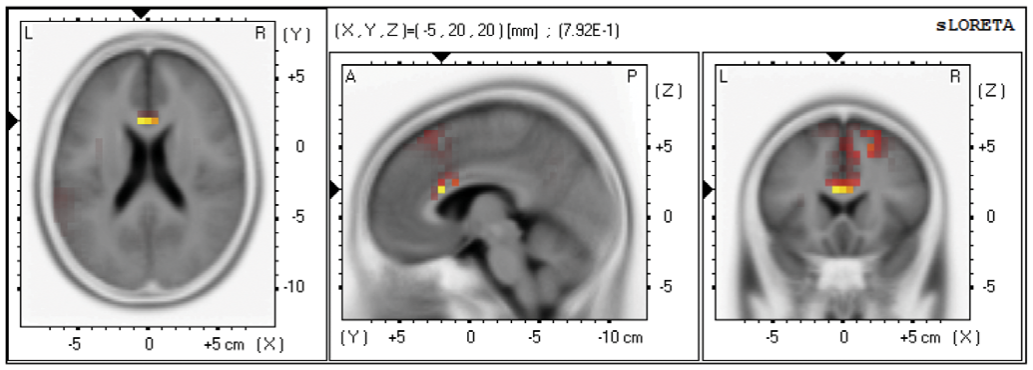
sLORETA solutions on P3 temporal factor scores showing voxels in which the Nogo>Go contrast was significant (p<0.01).

The voxel-based whole-brain sLORETA images for Nogo and Go conditions were compared using non-parametric randomization tests in order to identify the cortical regions involved in response inhibition. As illustrated in Fig 4, significant sources relating to the Nogo-P3 are located in the ACC (x = −5, y = 20, z = 20; BA 24/32) and showed significantly greater activation during response inhibition (Nogo conditions) than during response execution (Go conditions). Although in topographic demonstrations of P3 difference waves, the scalp distribution of this component showed considerable difference between two groups, we failed to obtain any significant between-group difference in source analysis.

## Discussion

The present study examined the difference in influence of emotional contexts on response inhibition at the behavioral and neural levels between alexithymic and control groups. Results have shown no significant difference of RTs or commission errors and omission errors between the 2 groups at the behavioral level. However, in neural level, particularly in N2 and P3 stage, positive emotion elicited larger frontal N2 amplitude in both alexithymic and control groups and during the negatively valenced context alexithymic group elicited larger central P3 amplitudes in the Nogo–Go difference wave compared to the controls. These data suggest that in negative emotional context, the inhibition process shows lesser interference in the alexithymia subjects than in controls. Thus, our result provides further evidence at the neural level, that difficulty in identifying feelings is a major feature of alexithymia, particularly in negative emotion.

In our study, we found no significant difference of RTs or commission errors and omission errors between two groups at the behavioral level. Nevertheless, using ERP, we discovered the significant different influence of negative emotional context on response inhibition between alexithymic and control groups at the neural level. To that extent, behavioral methods do not precisely reflect the differences of emotional contexts modulating response inhibition. Thus, we considered that it’s necessary to use the method of ERP as a more delicate way to examine the different influence of emotional contexts on response inhibition function of alexithymia.

The figure 2 showed that the inhibition of prepotent responses during the positively valenced context elicited larger frontal Nogo-N2 amplitudes than the other two emotional contexts. These data indicated that positive situations may cause a great deal of conflict. Our current result corresponds with previous findings that positive emotion impaired visual selective attention by increasing processing of spatially adjacent flanking distracters due to an increase in the scope of visuospatial attention [34]. Also, the “broaden-and-build” theory put forward that positive emotions broaden momentary thought-action repertories due to the deficit of inhibitory function [35]. In this regard, we considered that emotional contexts could modulate individual’s behavior. Particularly, positive emotion could enhance the scope of individual’s attention by reducing the functionality of inhibitory control mechanisms.

In current study, the Nogo–Go P3 occurring between 450–550 ms was larger during negative emotional context than during the other 2 emotional contexts. Furthermore, the source-localization experiment implicated the ACC as the neural generator of the Nogo-P3. These findings were congruent with some previous neuroimaging studies [36]. Some electrophysiological studies also have suggested that the decreased amplitude of the Nogo-P3 relates to the reduced inhibition ability [37], [38]. While other studies including our previous findings suggested that alexithymic individuals have deficits in fast conflict processing of information in the attention network test [39]. In addition, alexithymic individuals experienced greater interference, as indicated by their total interference score on the Stroop task, and were significantly slower than subjects with low TAS in completing the interference condition [40]. The preserved inhibitory control in emotional context can be interpreted by taking the core characteristic of alexithymia into account. Difficulty in identifying one’s emotional state is a major feature of alexithymia. Prkachin [41] found that alexithymic individuals specifically manifest difficulties in detecting negative emotions, such as anger, sadness, and fear, by investigating the ability of detecting and rating the intensity of emotional facial expressions. Functional neuroimaging studies have reported that alexithymic individuals exhibit decreased activation in the dorsal ACC in response to painful pictures [25] and low spontaneous reactivity of the amygdala to sad faces [42]. These findings indicated that less processing of negative emotion in alexithymic groups could generate less interference in the inhibition stages in the negative emotional context. Thus, in our study, it is not surprising that P3 amplitudes of the Nogo–Go difference wave during negative emotional context for alexithymic subjects was larger than that for controls. Our findings supported the hypothesis that alexithymia involves difficulty in identifying one’s emotional state, particularly negative emotions. Evolution has considered that negative emotions are one of the signals to identify potential threat in environment [43]. Moreover, negative emotions could prompt autonomic reﬂex actions and motor responses acted directly to counter threats and escape punishment, namely, adjust behaviors to adapt to the changing environment [44]. Our result suggested that alexithymic individuals may have potential obstacle of behavioral adaptation in the dangerous environment which can serve as a crucial functional impairment in a brand range of psychiatric disorders.

A guiding principle about ACC function is that cognitive and emotional information are processed separately. Its 2 major subdivisions, a dorsal cognitive division and a rostral–ventral affective division, serve distinct functions [45]. There is direct evidence that alexithymia, a personality trait important in affect regulation, is linked with the ACC during emotional stimuli processing [24] and response to emotion cues [46]. Additionally, the ACC plays a central role in executive function, particularly in attention and inhibitory processes [47], [48]. The involvement of the ACC in inhibition and emotion interaction was supported by the evidences that showed ACC was related with response inhibition [49] and affective stimuli response [46] respectively. In our study, this interaction in alexithymia was most prominently reported at the intersection of ACC, which agreed with previous neuroimaging studies using emotional Nogo–Go tasks [19]. This result suggests that the ACC plays a critical role in emotion-modulated response inhibition related to alexithymia.

The concept of alexithymia was considered to be an enduring personality trait which has been supported by several studies on clinical and general populations [3], [50], [51]. This trait has been suggested to stably exist in either psychiatric or somatic patients [50], [52]. Nevertheless, the stability of alexithymia seems to be more consistent in general populations compared to clinical populations [51]. Thus, general populations study of alexithymia may provide more representative information about this trait itself, while the patients study could specifically concentrate on the relationship between the alexithymic trait and the vulnerability to mental symptoms. In future, the clinical investigation would be needed to determine the role of alexithymia in psychosomatic disorders.

In summary, our study proved that contextual inhibitory paradigm which elicits activation of inhibition-related neural circuitry may be a useful method to examine the interaction of emotion and response inhibition in alexithymic group. Moreover, our results verified in neural level that the difficulty of identifying emotional feelings, particularly in negative emotion, is a major feature of alexithymia. This feature suggested that alexithymic individuals were not able to adjust their behavioral decisions in flexible emotional contexts which may lead to a maladaptation to threat information in social circumstance. Future studies are required to clarify the role of the subdivisions of ACC in a more complicate interaction of response inhibition, emotional valence, and arousal.
